# Supplementary material for: Visualizing genomic characteristics across an RNA-Seq based reference landscape of normal and neoplastic brain
Source: Sci Rep. 2023 Mar 14;13:4228. doi: 10.1038/s41598-023-31180-z (PMC10014937; doi:10.1038/s41598-023-31180-z)
Supplement: Supplementary file 1 — Supplementary Figures. [file 41598_2023_31180_MOESM1_ESM.pptx]

## Slide 1
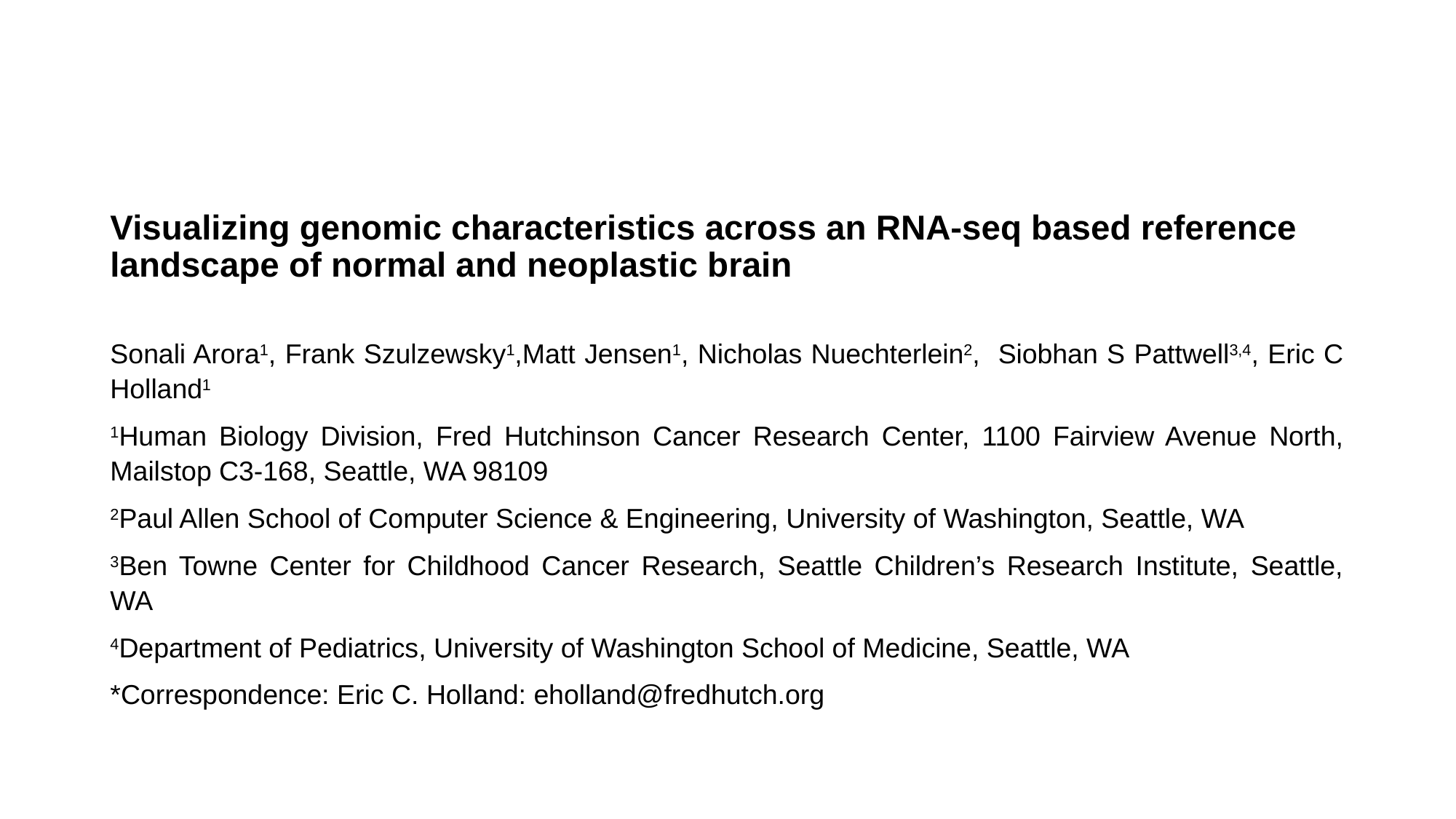

# Visualizing genomic characteristics across an RNA-seq based reference landscape of normal and neoplastic brain
Sonali Arora1, Frank Szulzewsky1,Matt Jensen1, Nicholas Nuechterlein2, Siobhan S Pattwell3,4, Eric C Holland1
1Human Biology Division, Fred Hutchinson Cancer Research Center, 1100 Fairview Avenue North, Mailstop C3-168, Seattle, WA 98109
2Paul Allen School of Computer Science & Engineering, University of Washington, Seattle, WA
3Ben Towne Center for Childhood Cancer Research, Seattle Children’s Research Institute, Seattle, WA
4Department of Pediatrics, University of Washington School of Medicine, Seattle, WA
*Correspondence: Eric C. Holland: eholland@fredhutch.org

## Slide 2
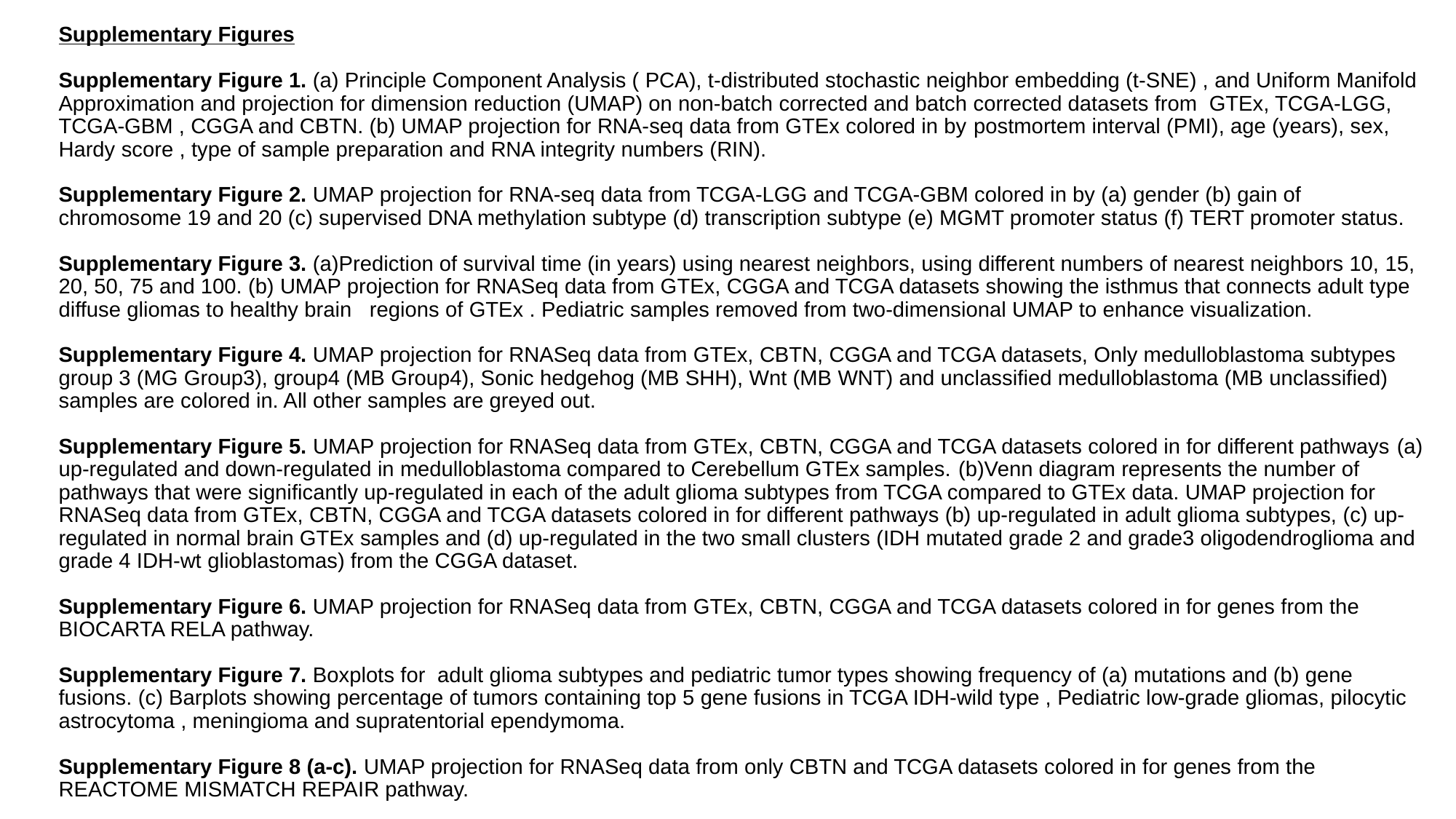

# Supplementary Figures Supplementary Figure 1. (a) Principle Component Analysis ( PCA), t-distributed stochastic neighbor embedding (t-SNE) , and Uniform Manifold Approximation and projection for dimension reduction (UMAP) on non-batch corrected and batch corrected datasets from GTEx, TCGA-LGG, TCGA-GBM , CGGA and CBTN. (b) UMAP projection for RNA-seq data from GTEx colored in by postmortem interval (PMI), age (years), sex, Hardy score , type of sample preparation and RNA integrity numbers (RIN). Supplementary Figure 2. UMAP projection for RNA-seq data from TCGA-LGG and TCGA-GBM colored in by (a) gender (b) gain of chromosome 19 and 20 (c) supervised DNA methylation subtype (d) transcription subtype (e) MGMT promoter status (f) TERT promoter status. Supplementary Figure 3. (a)Prediction of survival time (in years) using nearest neighbors, using different numbers of nearest neighbors 10, 15, 20, 50, 75 and 100. (b) UMAP projection for RNASeq data from GTEx, CGGA and TCGA datasets showing the isthmus that connects adult type diffuse gliomas to healthy brain regions of GTEx . Pediatric samples removed from two-dimensional UMAP to enhance visualization. Supplementary Figure 4. UMAP projection for RNASeq data from GTEx, CBTN, CGGA and TCGA datasets, Only medulloblastoma subtypes group 3 (MG Group3), group4 (MB Group4), Sonic hedgehog (MB SHH), Wnt (MB WNT) and unclassified medulloblastoma (MB unclassified) samples are colored in. All other samples are greyed out. Supplementary Figure 5. UMAP projection for RNASeq data from GTEx, CBTN, CGGA and TCGA datasets colored in for different pathways (a) up-regulated and down-regulated in medulloblastoma compared to Cerebellum GTEx samples. (b)Venn diagram represents the number of pathways that were significantly up-regulated in each of the adult glioma subtypes from TCGA compared to GTEx data. UMAP projection for RNASeq data from GTEx, CBTN, CGGA and TCGA datasets colored in for different pathways (b) up-regulated in adult glioma subtypes, (c) up-regulated in normal brain GTEx samples and (d) up-regulated in the two small clusters (IDH mutated grade 2 and grade3 oligodendroglioma and grade 4 IDH-wt glioblastomas) from the CGGA dataset. Supplementary Figure 6. UMAP projection for RNASeq data from GTEx, CBTN, CGGA and TCGA datasets colored in for genes from the BIOCARTA RELA pathway. Supplementary Figure 7. Boxplots for adult glioma subtypes and pediatric tumor types showing frequency of (a) mutations and (b) gene fusions. (c) Barplots showing percentage of tumors containing top 5 gene fusions in TCGA IDH-wild type , Pediatric low-grade gliomas, pilocytic astrocytoma , meningioma and supratentorial ependymoma. Supplementary Figure 8 (a-c). UMAP projection for RNASeq data from only CBTN and TCGA datasets colored in for genes from the REACTOME MISMATCH REPAIR pathway.

## Slide 3
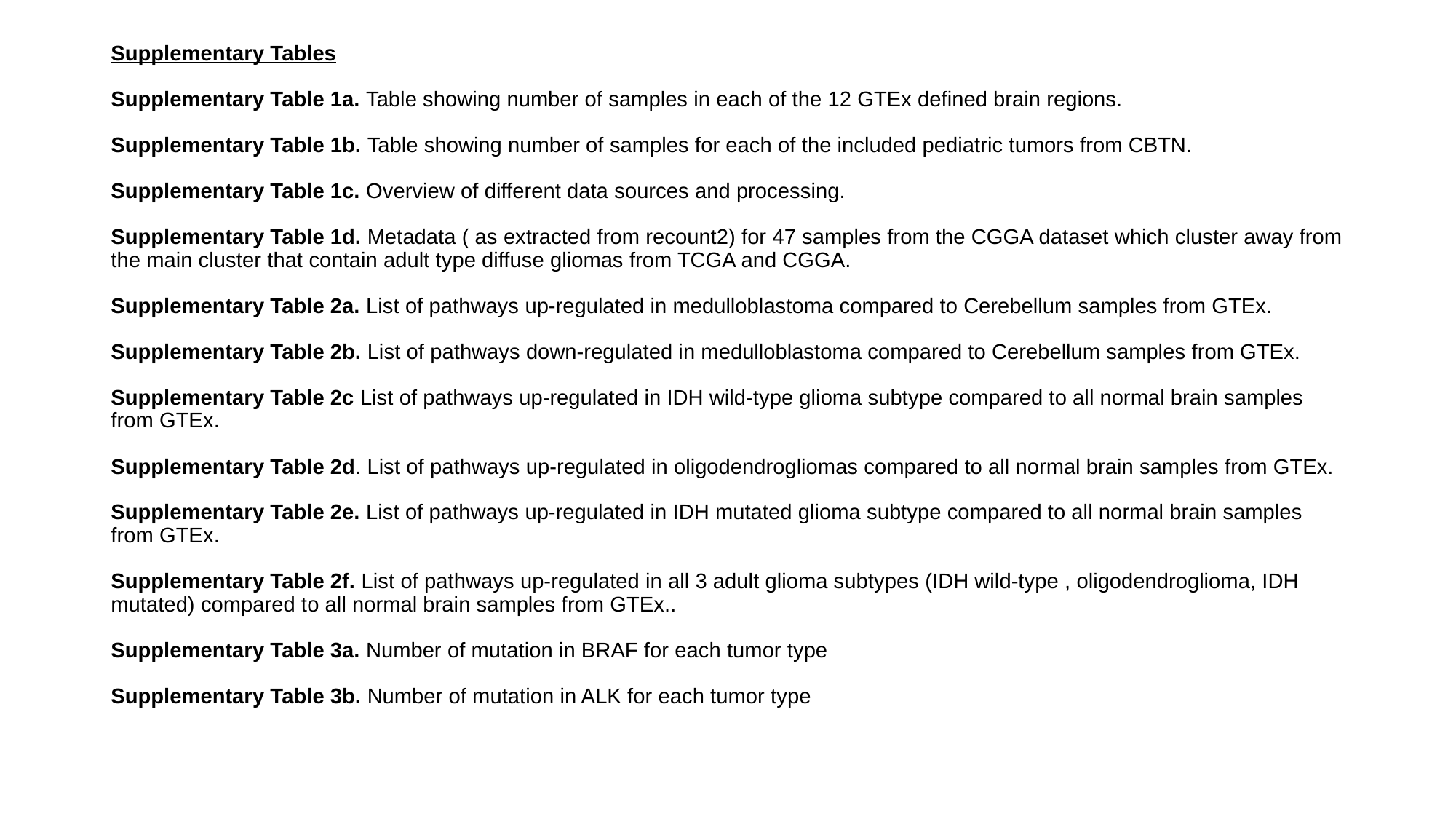

# Supplementary Tables Supplementary Table 1a. Table showing number of samples in each of the 12 GTEx defined brain regions. Supplementary Table 1b. Table showing number of samples for each of the included pediatric tumors from CBTN. Supplementary Table 1c. Overview of different data sources and processing. Supplementary Table 1d. Metadata ( as extracted from recount2) for 47 samples from the CGGA dataset which cluster away from the main cluster that contain adult type diffuse gliomas from TCGA and CGGA. Supplementary Table 2a. List of pathways up-regulated in medulloblastoma compared to Cerebellum samples from GTEx. Supplementary Table 2b. List of pathways down-regulated in medulloblastoma compared to Cerebellum samples from GTEx. Supplementary Table 2c List of pathways up-regulated in IDH wild-type glioma subtype compared to all normal brain samples from GTEx. Supplementary Table 2d. List of pathways up-regulated in oligodendrogliomas compared to all normal brain samples from GTEx. Supplementary Table 2e. List of pathways up-regulated in IDH mutated glioma subtype compared to all normal brain samples from GTEx. Supplementary Table 2f. List of pathways up-regulated in all 3 adult glioma subtypes (IDH wild-type , oligodendroglioma, IDH mutated) compared to all normal brain samples from GTEx.. Supplementary Table 3a. Number of mutation in BRAF for each tumor type Supplementary Table 3b. Number of mutation in ALK for each tumor type

## Slide 4
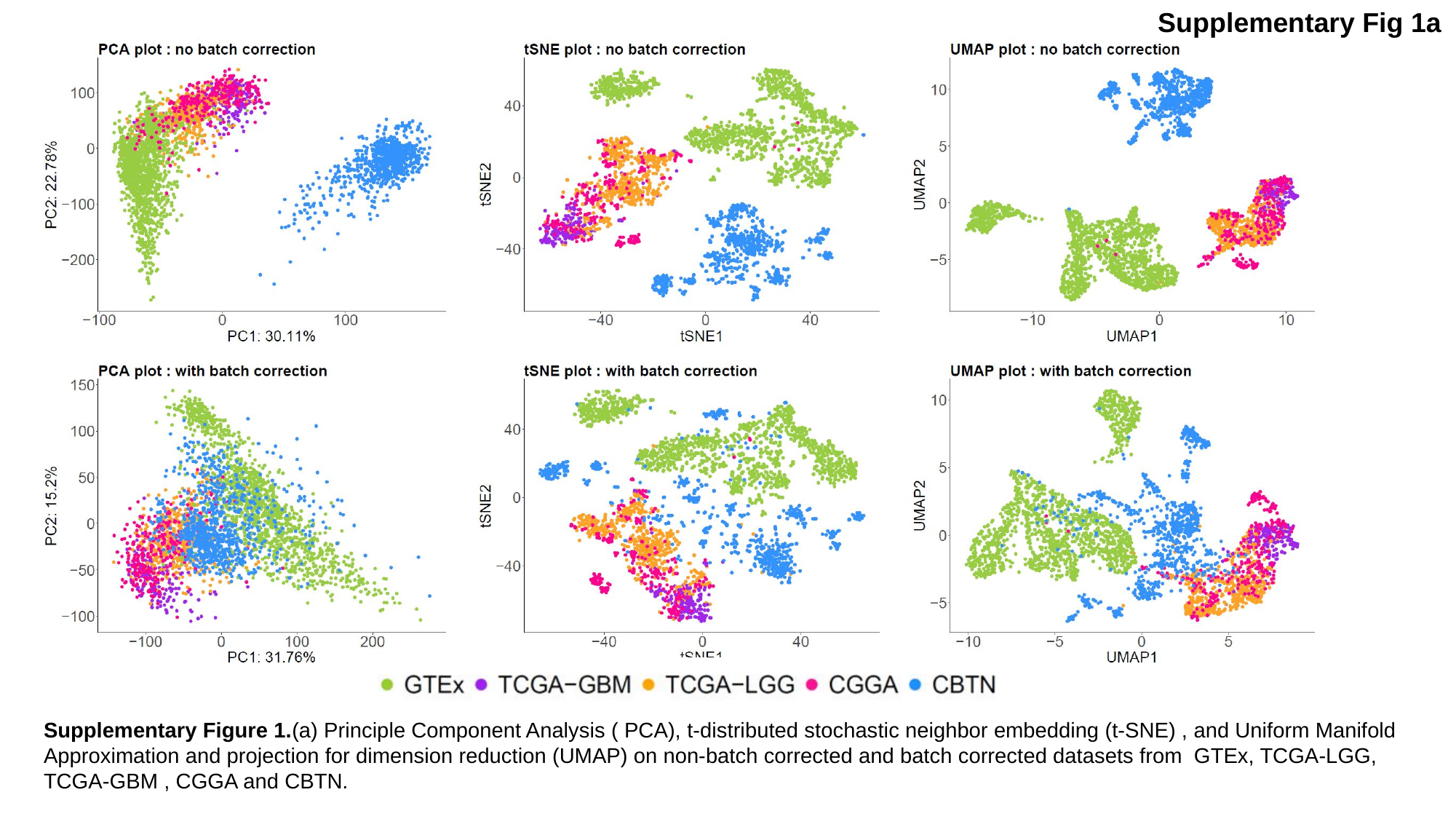

Supplementary Fig 1a
Supplementary Figure 1.(a) Principle Component Analysis ( PCA), t-distributed stochastic neighbor embedding (t-SNE) , and Uniform Manifold Approximation and projection for dimension reduction (UMAP) on non-batch corrected and batch corrected datasets from GTEx, TCGA-LGG, TCGA-GBM , CGGA and CBTN.

## Slide 5
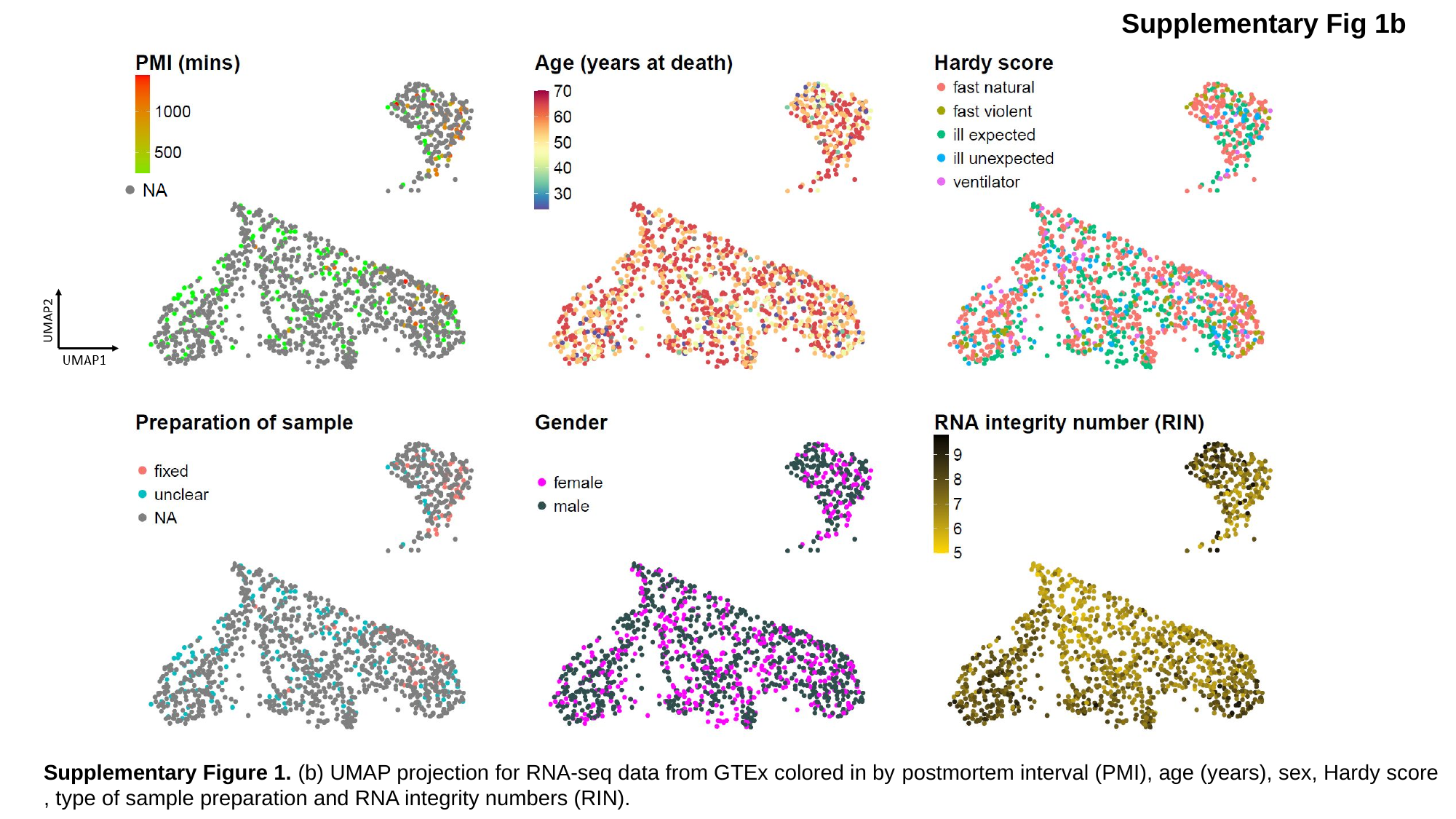

Supplementary Fig 1b
Supplementary Figure 1. (b) UMAP projection for RNA-seq data from GTEx colored in by postmortem interval (PMI), age (years), sex, Hardy score , type of sample preparation and RNA integrity numbers (RIN).

## Slide 6
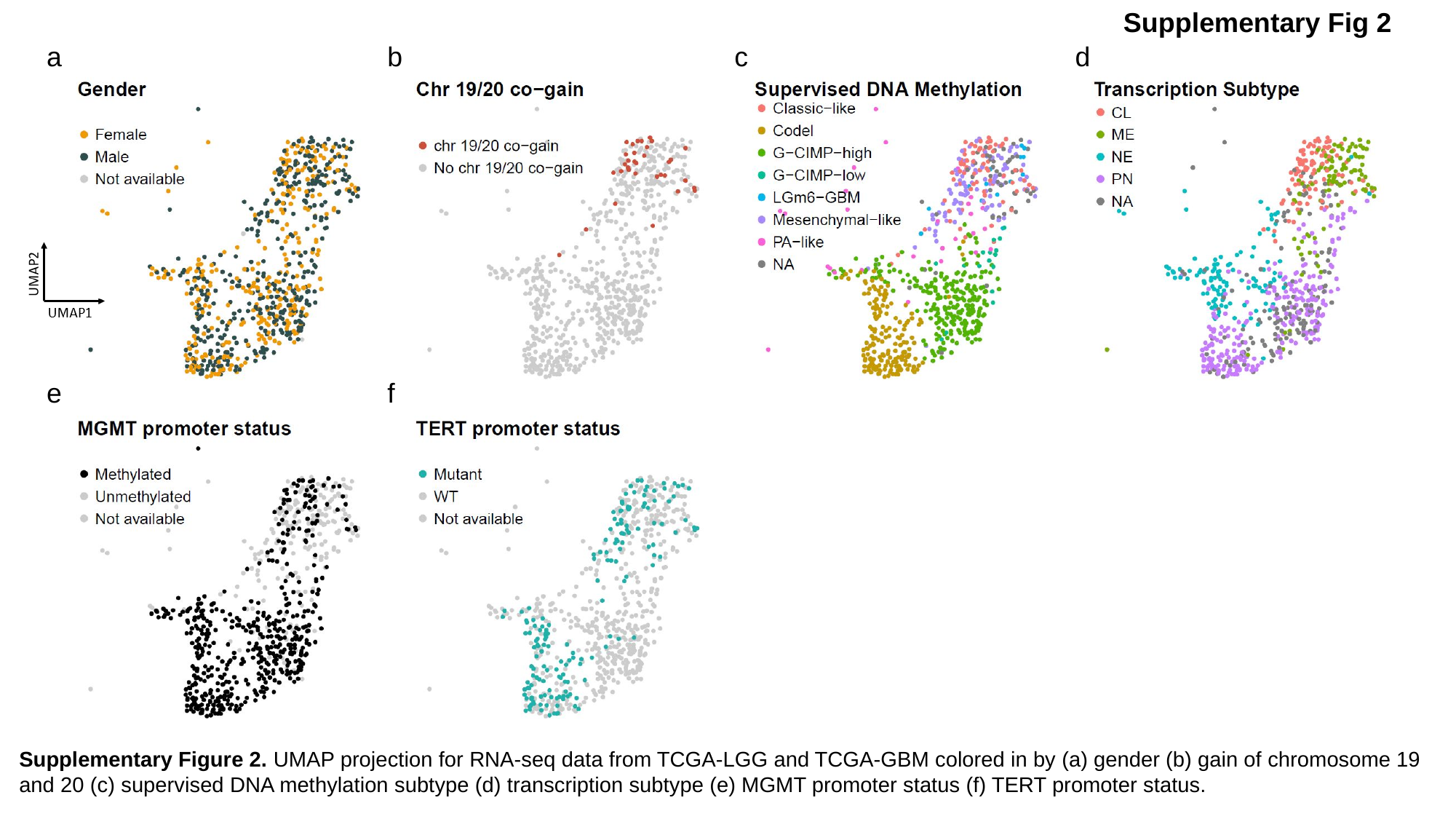

Supplementary Fig 2
a
b
c
d
e
f
Supplementary Figure 2. UMAP projection for RNA-seq data from TCGA-LGG and TCGA-GBM colored in by (a) gender (b) gain of chromosome 19 and 20 (c) supervised DNA methylation subtype (d) transcription subtype (e) MGMT promoter status (f) TERT promoter status.

## Slide 7
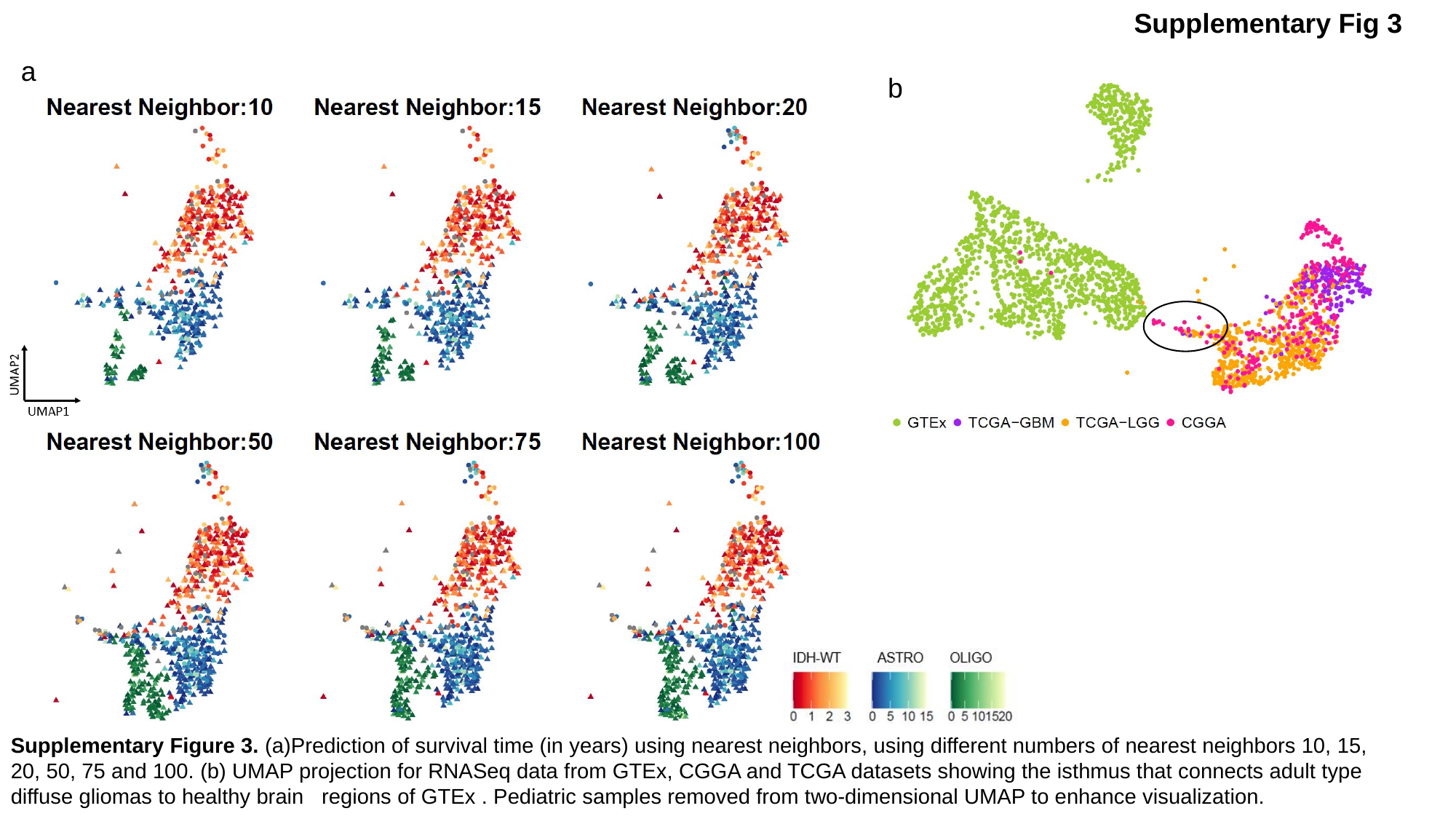

Supplementary Fig 3
a
b
Supplementary Figure 3. (a)Prediction of survival time (in years) using nearest neighbors, using different numbers of nearest neighbors 10, 15, 20, 50, 75 and 100. (b) UMAP projection for RNASeq data from GTEx, CGGA and TCGA datasets showing the isthmus that connects adult type diffuse gliomas to healthy brain regions of GTEx . Pediatric samples removed from two-dimensional UMAP to enhance visualization.

## Slide 8
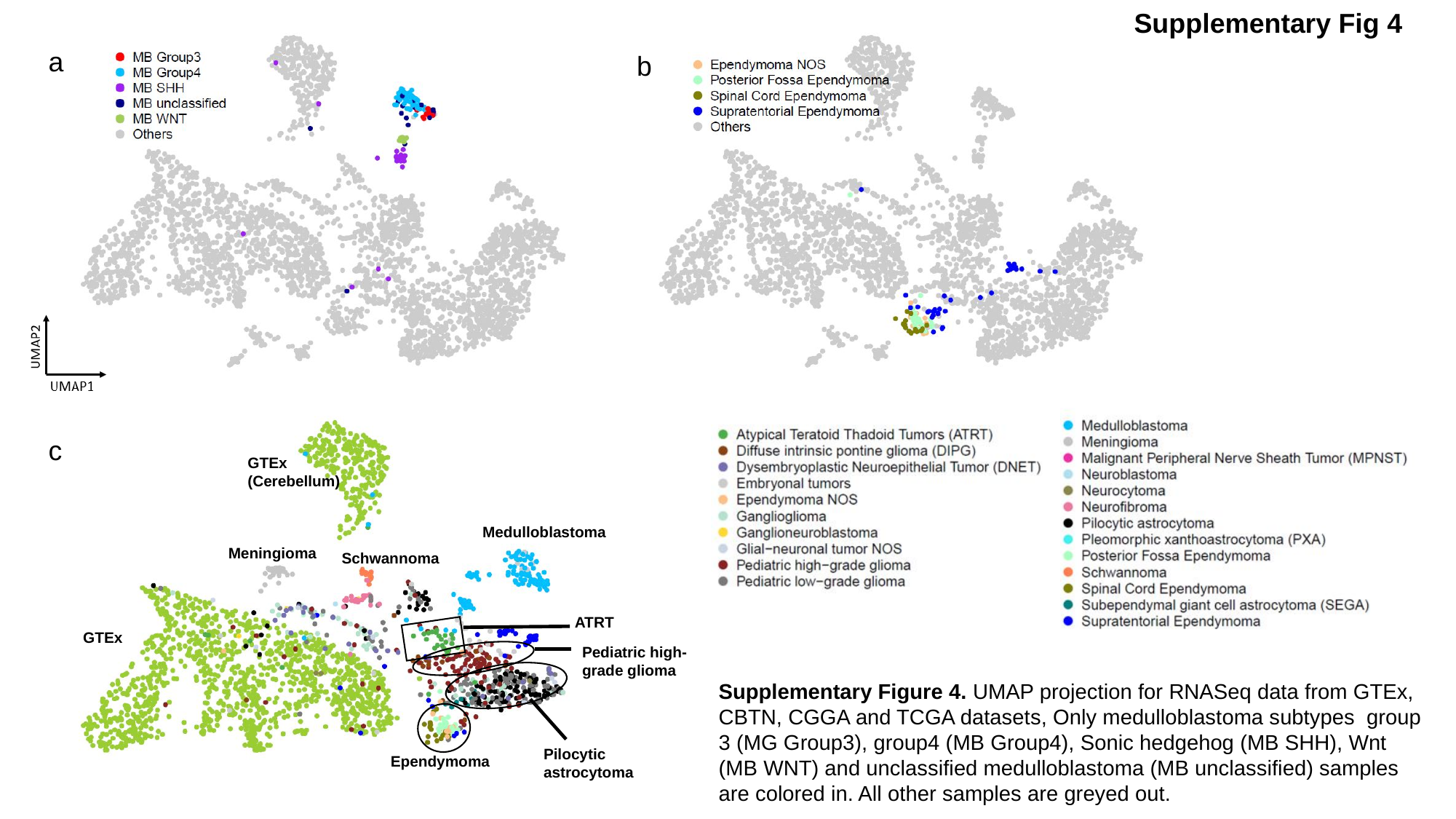

Supplementary Fig 4
a
b
c
GTEx
(Cerebellum)
Medulloblastoma
Meningioma
Schwannoma
ATRT
GTEx
Pediatric high-grade glioma
Supplementary Figure 4. UMAP projection for RNASeq data from GTEx, CBTN, CGGA and TCGA datasets, Only medulloblastoma subtypes group 3 (MG Group3), group4 (MB Group4), Sonic hedgehog (MB SHH), Wnt (MB WNT) and unclassified medulloblastoma (MB unclassified) samples are colored in. All other samples are greyed out.
Pilocytic astrocytoma
Ependymoma

## Slide 9
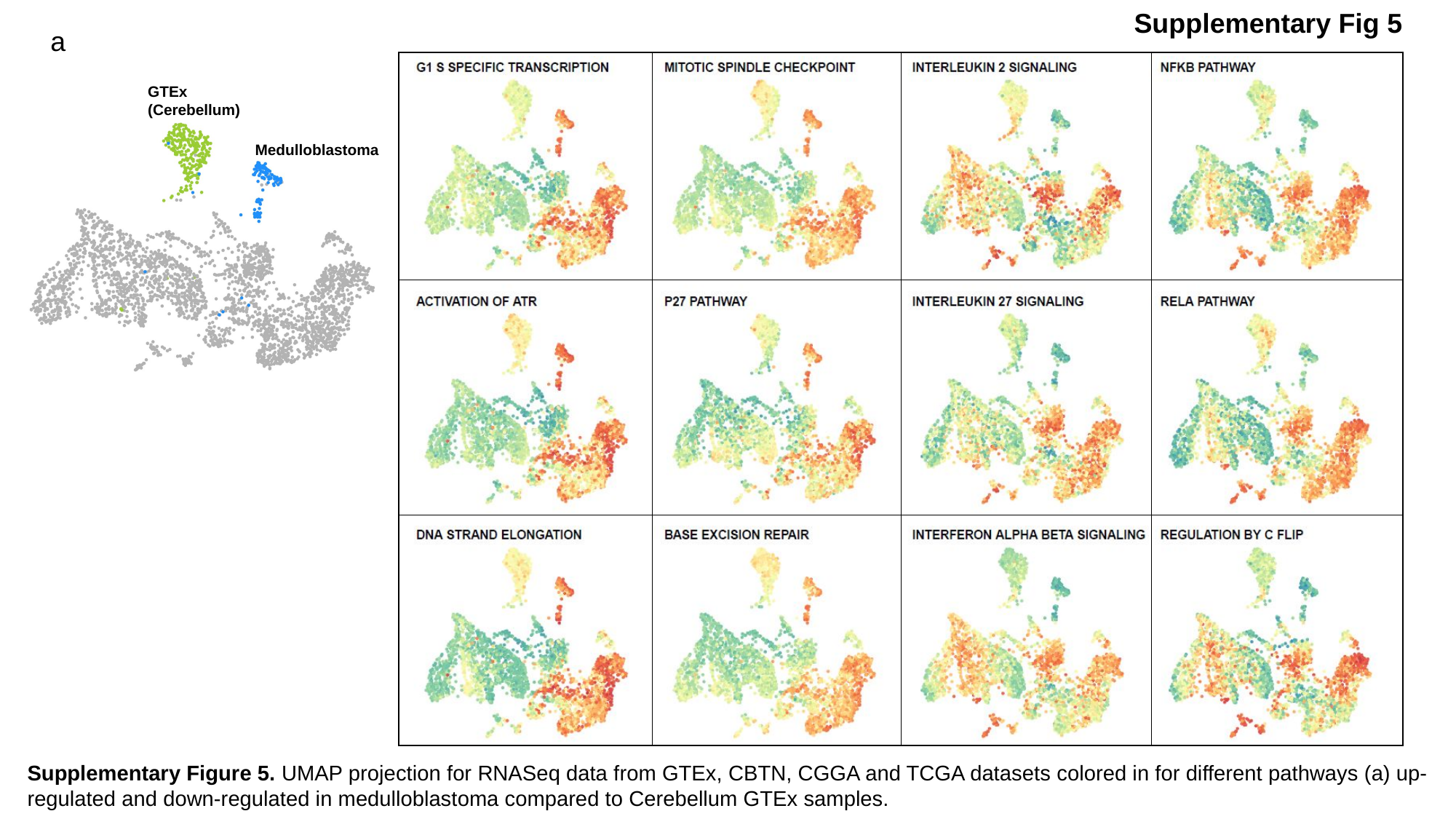

Supplementary Fig 5
a
GTEx
(Cerebellum)
Medulloblastoma
Supplementary Figure 5. UMAP projection for RNASeq data from GTEx, CBTN, CGGA and TCGA datasets colored in for different pathways (a) up-regulated and down-regulated in medulloblastoma compared to Cerebellum GTEx samples.

## Slide 10
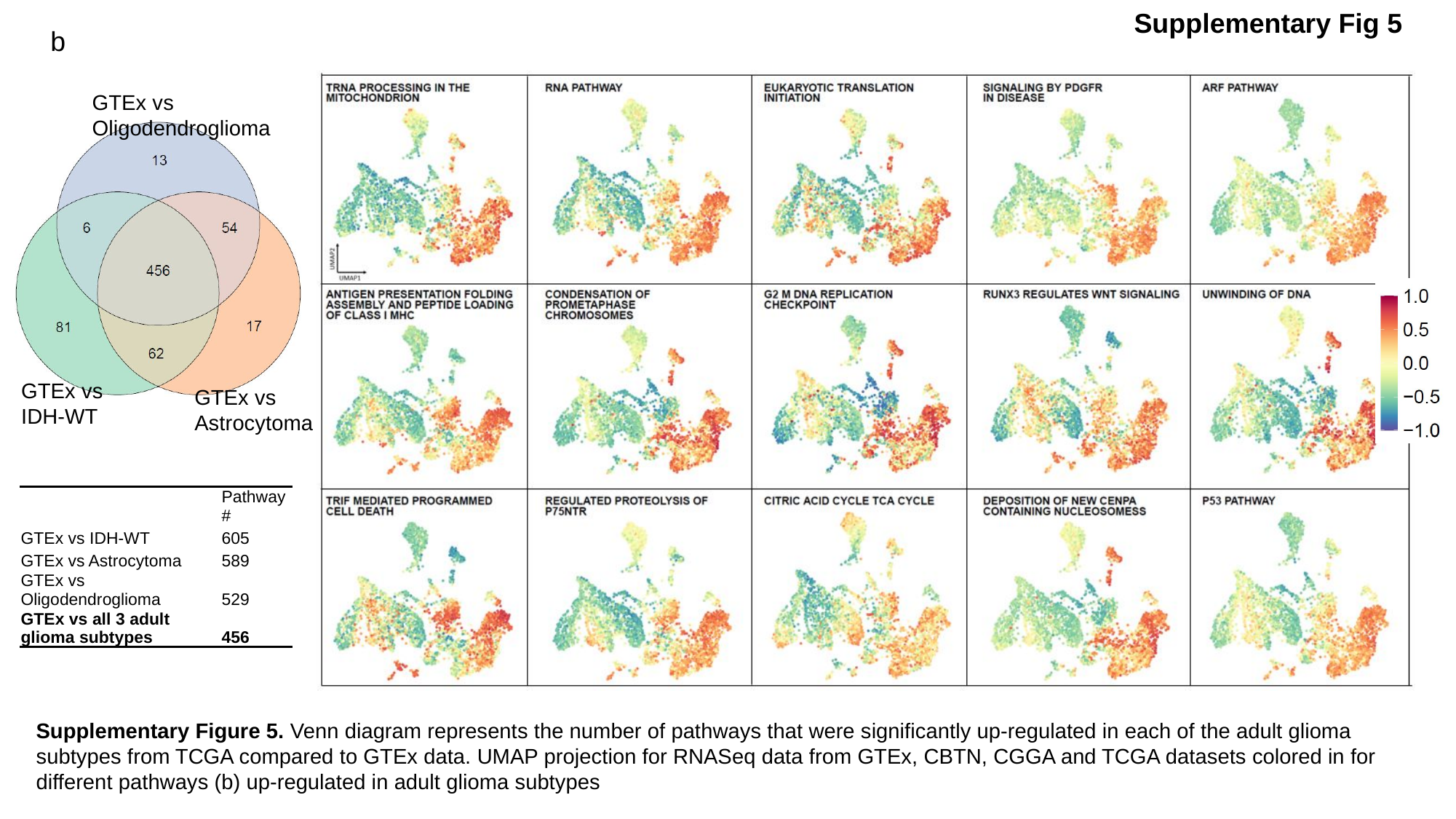

Supplementary Fig 5
b
GTEx vs Oligodendroglioma
GTEx vs
IDH-WT
GTEx vs
Astrocytoma
| | Pathway# |
| --- | --- |
| GTEx vs IDH-WT | 605 |
| GTEx vs Astrocytoma | 589 |
| GTEx vs Oligodendroglioma | 529 |
| GTEx vs all 3 adult glioma subtypes | 456 |
Supplementary Figure 5. Venn diagram represents the number of pathways that were significantly up-regulated in each of the adult glioma subtypes from TCGA compared to GTEx data. UMAP projection for RNASeq data from GTEx, CBTN, CGGA and TCGA datasets colored in for different pathways (b) up-regulated in adult glioma subtypes

## Slide 11
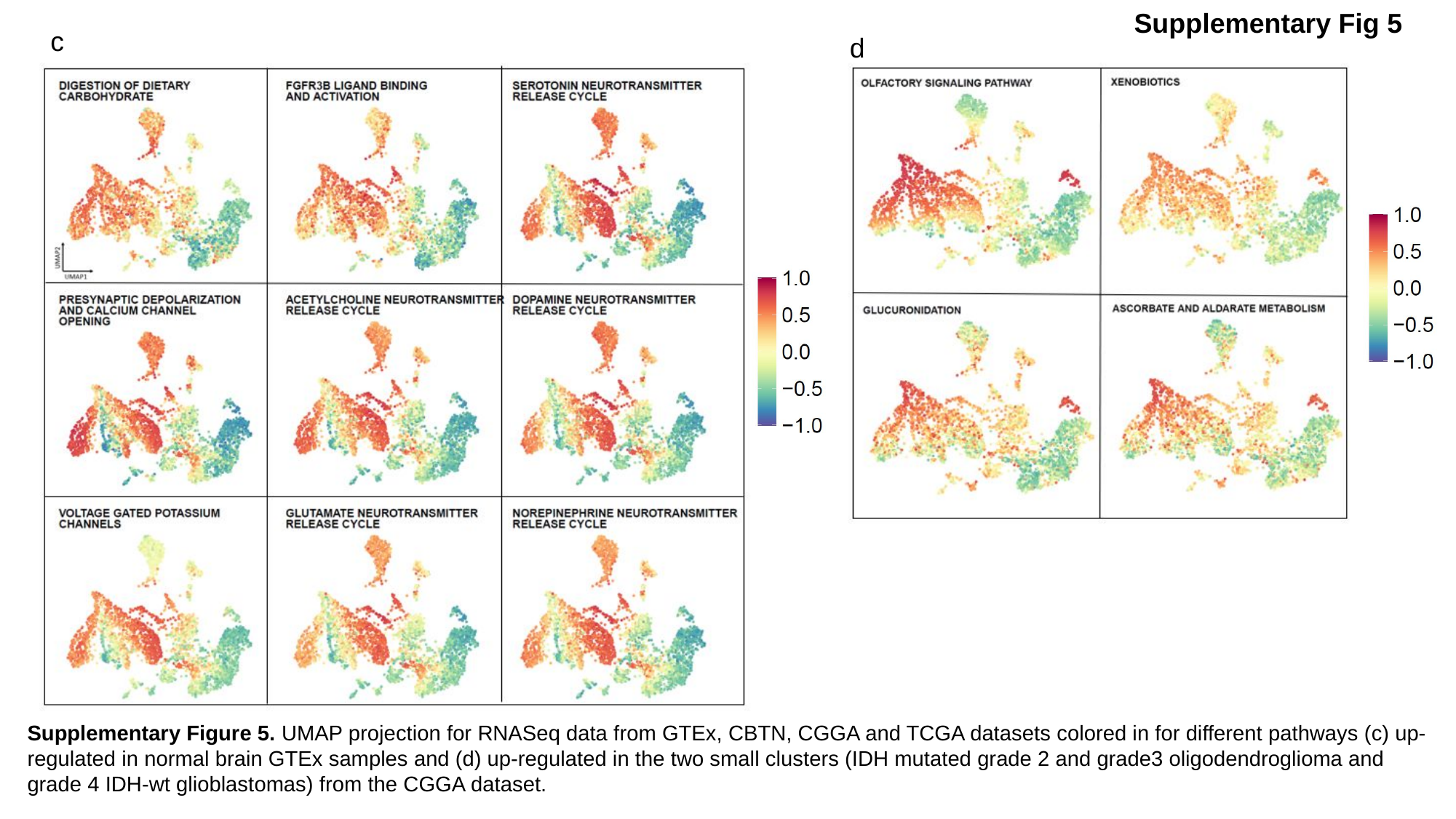

Supplementary Fig 5
c
d
Supplementary Figure 5. UMAP projection for RNASeq data from GTEx, CBTN, CGGA and TCGA datasets colored in for different pathways (c) up-regulated in normal brain GTEx samples and (d) up-regulated in the two small clusters (IDH mutated grade 2 and grade3 oligodendroglioma and grade 4 IDH-wt glioblastomas) from the CGGA dataset.

## Slide 12
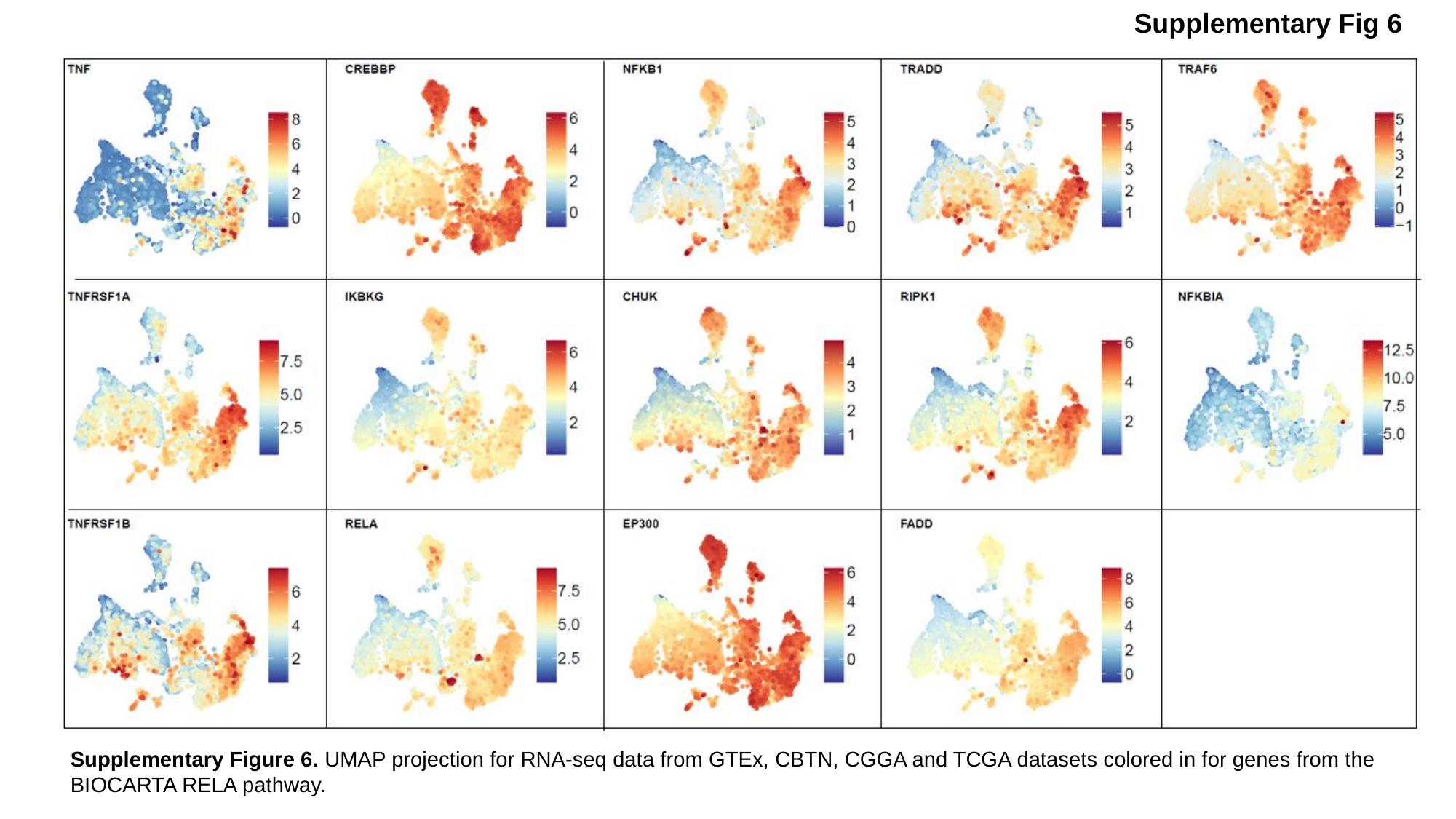

Supplementary Fig 6
Supplementary Figure 6. UMAP projection for RNA-seq data from GTEx, CBTN, CGGA and TCGA datasets colored in for genes from the BIOCARTA RELA pathway.

## Slide 13
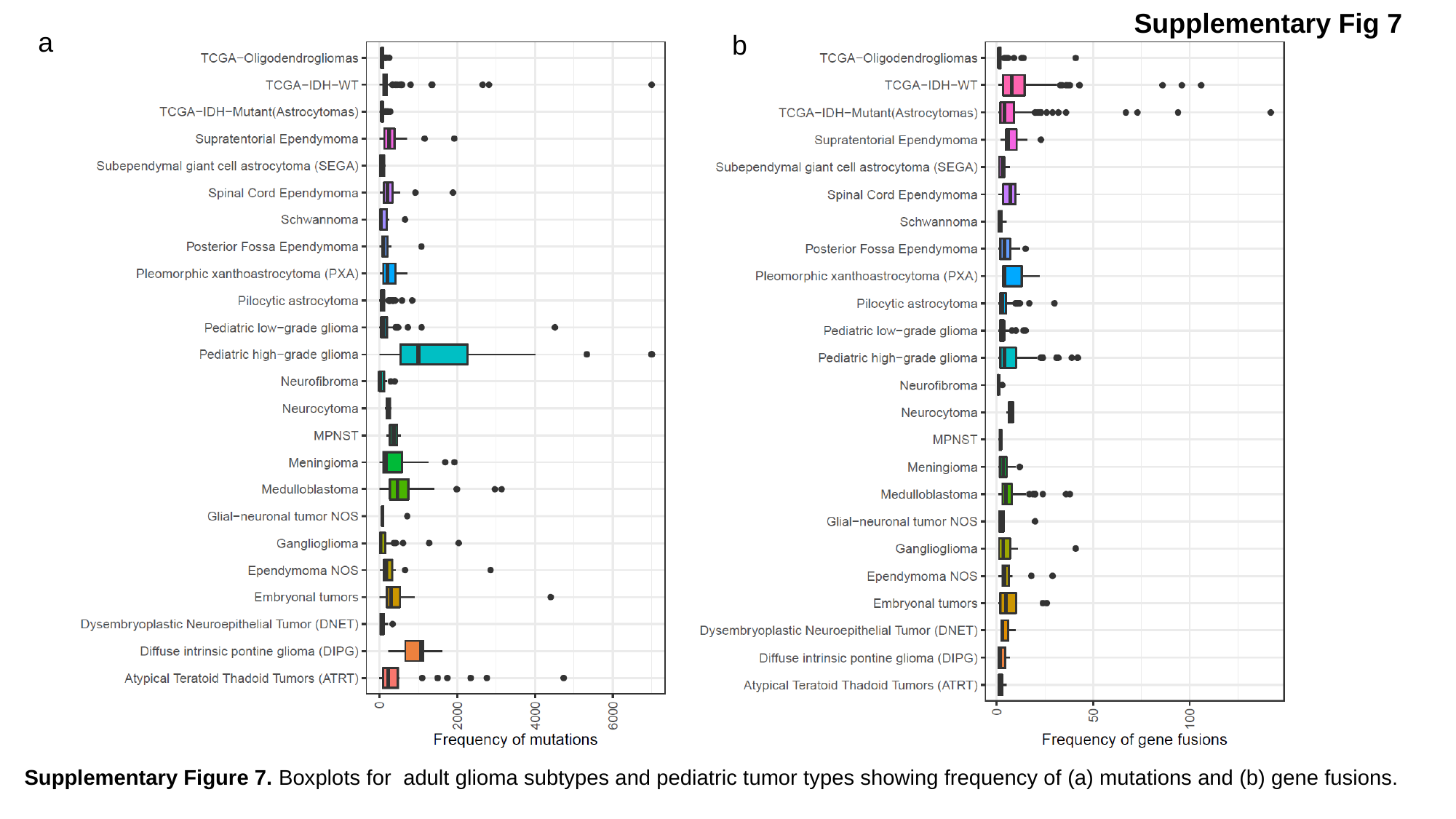

Supplementary Fig 7
a
b
Supplementary Figure 7. Boxplots for adult glioma subtypes and pediatric tumor types showing frequency of (a) mutations and (b) gene fusions.

## Slide 14
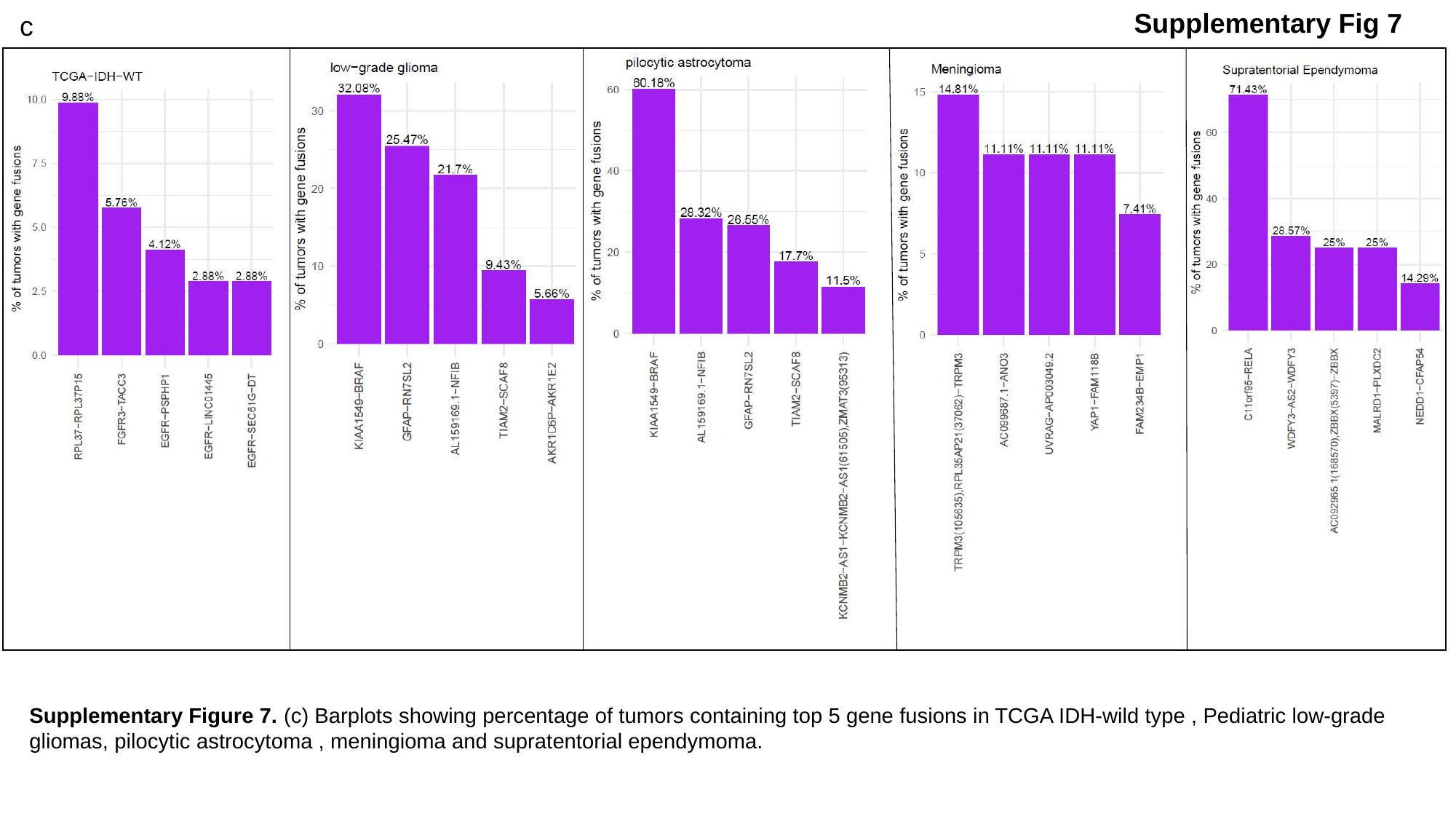

Supplementary Fig 7
c
Supplementary Figure 7. (c) Barplots showing percentage of tumors containing top 5 gene fusions in TCGA IDH-wild type , Pediatric low-grade gliomas, pilocytic astrocytoma , meningioma and supratentorial ependymoma.

## Slide 15
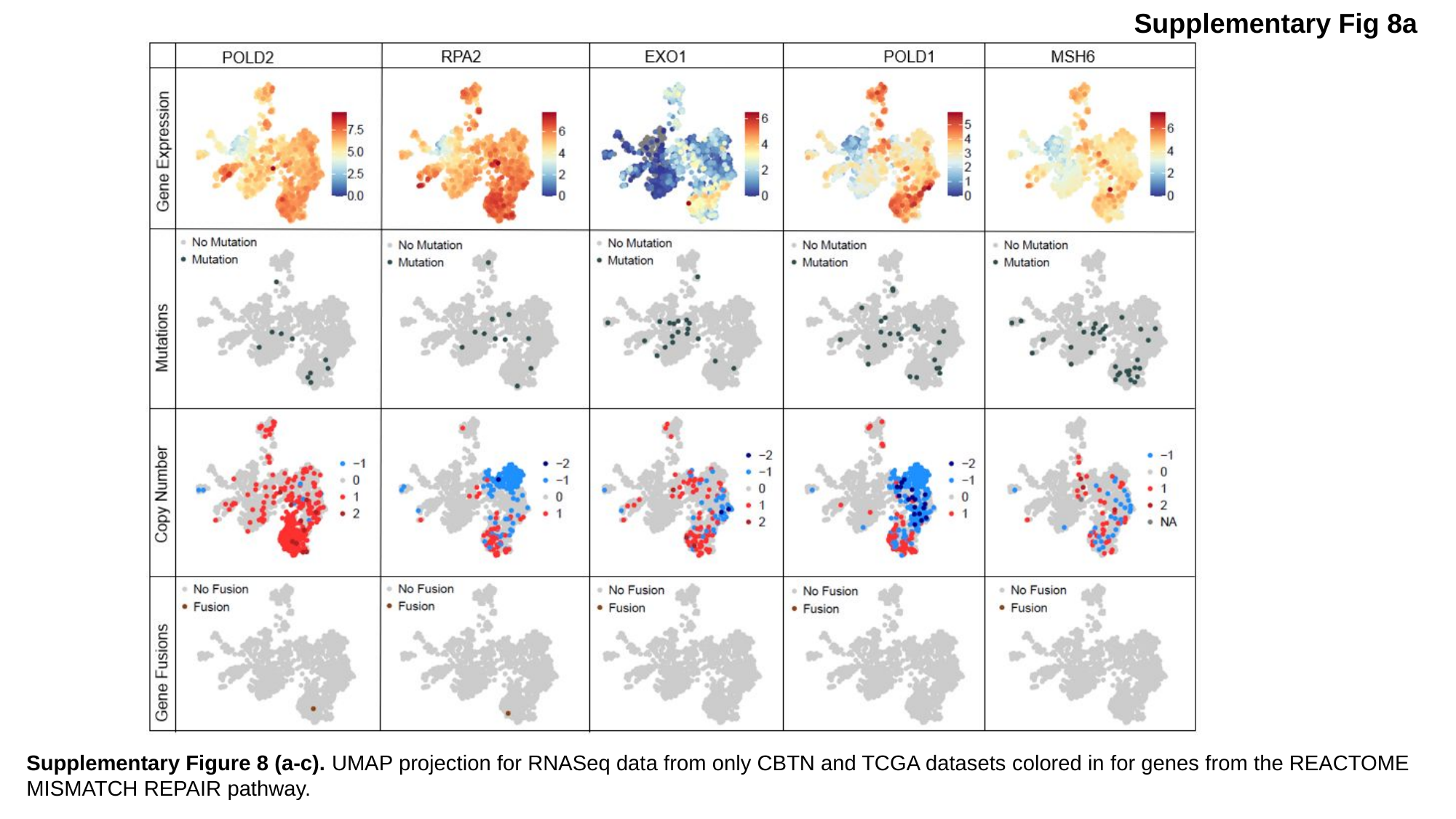

Supplementary Fig 8a
Supplementary Figure 8 (a-c). UMAP projection for RNASeq data from only CBTN and TCGA datasets colored in for genes from the REACTOME MISMATCH REPAIR pathway.

## Slide 16
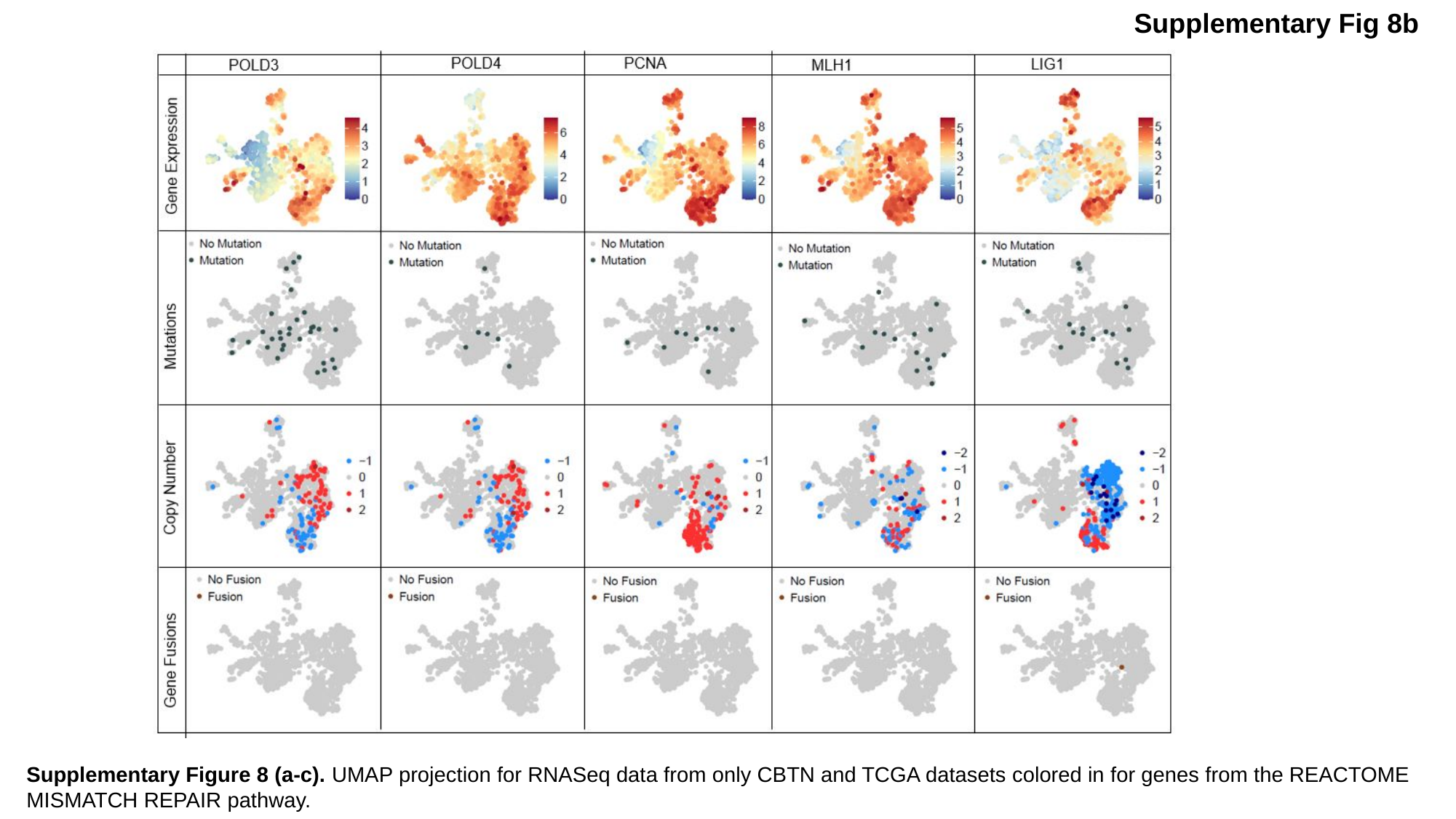

Supplementary Fig 8b
Supplementary Figure 8 (a-c). UMAP projection for RNASeq data from only CBTN and TCGA datasets colored in for genes from the REACTOME MISMATCH REPAIR pathway.

## Slide 17
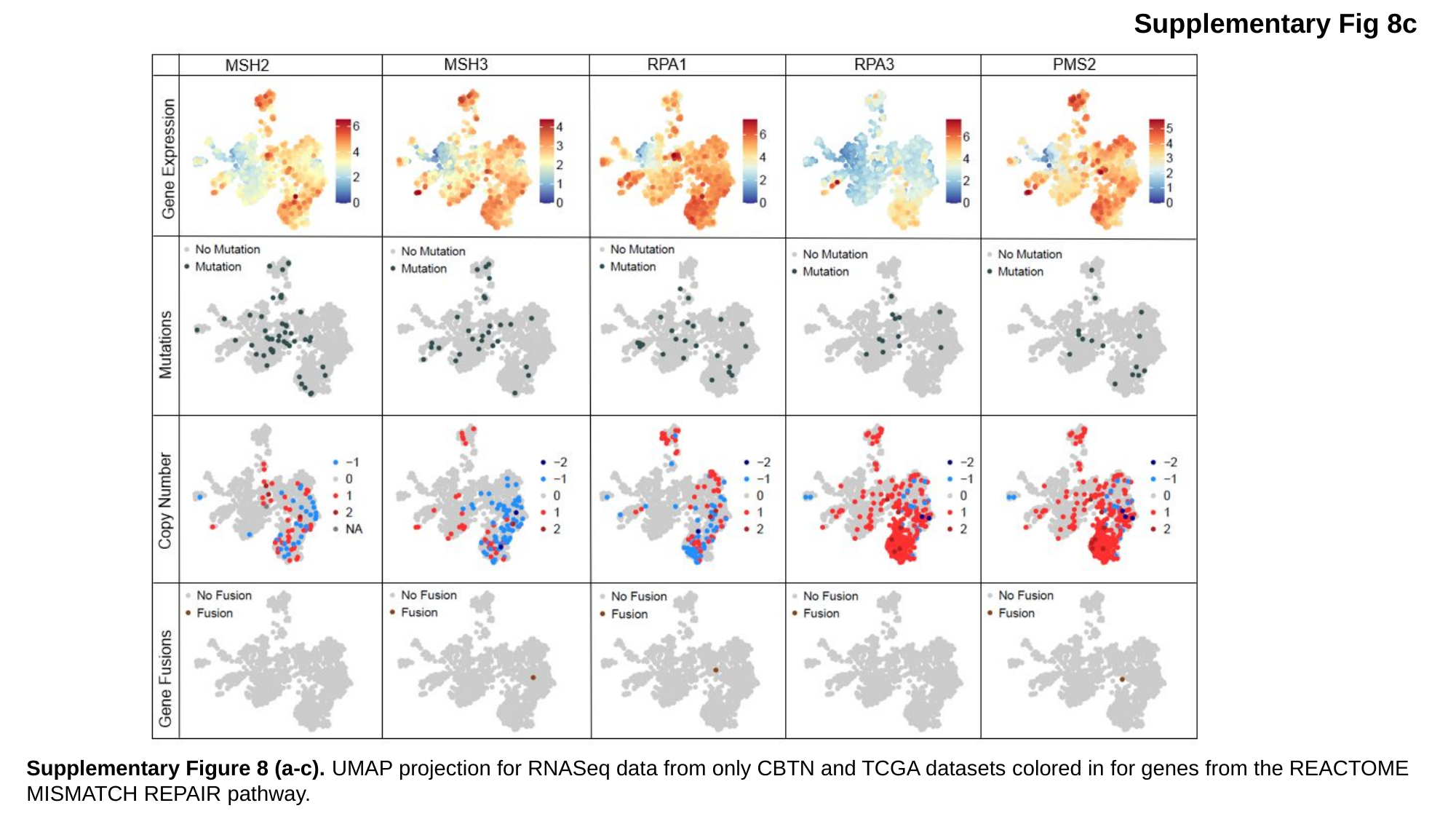

Supplementary Fig 8c
Supplementary Figure 8 (a-c). UMAP projection for RNASeq data from only CBTN and TCGA datasets colored in for genes from the REACTOME MISMATCH REPAIR pathway.
